# Clonal integration of stress signal induces morphological and physiological response of root within clonal network

**DOI:** 10.1371/journal.pone.0298258

**Published:** 2024-03-06

**Authors:** Su-Juan Duan, Jie Du, Dong-Wei Yu, Xiang-Jun Pei, Da-Qiu Yin, Shi-Jun Wang, Qi-Zhong Tao, Yi Dan, Xiao-Chao Zhang, Jie Deng, Jin-Song Chen, Qing Wei, Ning-Fei Lei

**Affiliations:** 1 College of Life Science, Sichuan Normal University, Chengdu, China; 2 Jiuzhaigou National Nature Reserve Administration, Sichuan, China; 3 College of Ecology and Environment, Chengdu University of Technology, Chengdu, China; 4 Huaneng Tibet Yarlung Zangbo River Hydropower Development and Investment Co., Ltd, Lhasa, China; 5 College of Pastoral Agricultural Science and Technology, State Key Laboratory of Grassland Agro-ecosystems, Lanzhou University, Lanzhou, China; 6 State Key Laboratory of Geohazard Prevention and Geoenvironment Protection, Chengdu University of Technology, Chengdu, China; Kohat University of Science and Technology, PAKISTAN

## Abstract

Clonal integration of defense or stress signal induced systemic resistance in leaf of interconnected ramets. However, similar effects of stress signal in root are poorly understood within clonal network. Clonal fragments of *Centella asiaticas* with first-young, second-mature, third-old and fourth-oldest ramets were used to investigate transportation or sharing of stress signal among interconnected ramets suffering from low water availability. Compared with control, oxidative stress in root of the first-young, second-mature and third-old ramets was significantly alleviated by exogenous ABA application to the fourth-oldest ramets as well as enhancement of antioxidant enzyme (SOD, POD, CAT and APX) activities and osmoregulation ability. Surface area and volume in root of the first-young ramets were significantly increased and total length in root of the third-old ramets was significantly decreased. POD activity in root of the fourth-oldest and third-old ramets was significantly enhanced by exogenous ABA application to the first-young ramets. Meanwhile, total length and surface area in root of the fourth-oldest and third-old ramets were significantly decreased. Ratio of belowground to aboveground biomass in the whole clonal fragments was significantly increased by exogenous ABA application to the fourth-oldest or first-young ramets. It is suggested that transportation or sharing of stress signal may induce systemic resistance in root of interconnected ramets. Specially, transportation or sharing of stress signal against phloem flow was observed in the experiment. Possible explanation is that rapid recovery of foliar photosynthesis in first-young ramets subjected to exogenous ABA application can partially reverse phloem flow within clonal network. Thus, our experiment provides insight into ecological implication on clonal integration of stress signal.

## Introduction

Clonal plants often propagate genetically identical ramets connected by spacer (stolon or rhizome) [1–3]. Resource substance (such as carbohydrates, nutrient and water) can be transported and shared among interconnected ramets, which is named as clonal integration [4–6]. So clonal integration may be very important for clonal plant, by provisioning internal support to ramets growing in patches of low resource availability or stressed environment [7–9].

Based on vascular connection such as stolon or rhizome, non-resource substance (such as defense signal) was transported or shared among interconnected ramets as well as resource substance [10–12]. With decrease of soluble carbohydrate content and change of phenolic composition, leaf strength and thickness of undamaged ramets were significantly increased by clonal integration of defense signal when interconnected ramets of stoloniferous herb *Trifolium repens* were subjected to herbivory damage from caterpillar *Spodoptera exigua* [13]. With increase of condensed tannin content, herbivory damage to leaf of young ramets was significantly alleviated by clonal integration of defense signal when jasmonic-acid was applied to the interconnected oldest ramets of rhizomatous sedge *Carex alrofusca* [14]. According to a risk-spreading strategy, systematic defense induced by clonal integration equalized herbivory preference and avoided selective feeding on young plant tissues [15]. With increase of antioxidant enzyme (SOD, POD, CAT and APX) activities, oxidative stress (O_2_^•−^ production rate and malondialdehyde content) in the leaf of the old, mature and young ramets was significantly alleviated by exogenous ABA application to the oldest ramets of stoloniferous herb *Centella asiaticas* subjected to low water availability [16]. Generally, clonal integration of defense or stress signal may be dependent on phloem flow driven by source-sink relationship [16–18].

Abscisic acid plays an very important role in the plant response to water deficit [19]. Exogenous ABA application improved leaf photosynthetic rate and water use efficiency when the soybean was subjected to water deficit [20]. With exogenous ABA application, endogenous proline levels, chlorophyll, carotenoid, total carbohydrate contents, acid phosphatase and peroxidase were significantly increased when the *Pisum sativum* was subjected to water deficit [21]. To our knowledge, previous studies focused on foliar response of interconnected ramets induced by clonal integration of defense or stress signal. However, effects of transportation or sharing of defense or stress signal in root are poorly understood within clonal network. A pot experiment was conducted to investigate clonal integration of stress signal and its effects on morphological and physiological traits of root within clonal network.

As a perennial medicinal plant, *Centella asiatica* contain asiaticoside, madecasosside, asiatic acid and madecassic acid [22]. Clonal fragments of *Centella asiatica* first-young, second-mature, third-old, and fourth-oldest were suffering from low water availability (20% soil moisture content). Meanwhile, 5 mL ABA solution (0.1 mM) or same volume distilled water was applied to the first-young or fourth-oldest ramets respectively. Comparing with control (same volume distilled water treatment), we predicted that: (1) with increase of antioxidant enzyme (SOD, POD, CAT and APX) activities, oxidative stress (O_2_^•−^ production rate and H_2_O_2_ content) in root of the first-young, second-mature and third-old ramets was significantly alleviated by exogenous ABA application to the fourth-oldest ramets; (2) proline (pro) and soluble protein content in root of the first-young, second-mature and third-old ramets were significantly increased by exogenous ABA application to the fourth-oldest ramets; (3) biomass accumulation and ratio of belowground to aboveground biomass in whole clonal fragment were significantly increased by exogenous ABA application to the fourth-oldest ramets; (4) total length, surface area and volume in root of the first-young, second-mature and third-old ramets were significantly greater when exogenous ABA was applied the fourth-oldest ramets.

Within clonal network, transportation or sharing of defense or stress signal can be dependent on phloem flow driven by source-sink relationship [17, 23]. Finally, we predicted that similar patterns were not observed in root of the second-mature, third-old and fourth-oldest ramets when exogenous ABA was applied to the first-young ramets.

## Materials and methods

### Plant material

As a stoloniferous perennial herb, *Centella asiatica* (Umbelliferae) is distributed predominantly in woodlands, forests edges, damp grass, roadside or creek (about 200-1900m) [24]. Its stolon usually takes roots in contact with moist substratum, which forming a network of stolon above the ground. Each ramet of *C*. *asiatica* is consists of two zygomorphic leaves with slender petiole [25].

Seven original plants of *C*. *asiatica* were collected in Chengdu, Sichuan Province, China (30°05′-31°260N; 102°540–104°53°E). The original plants were away from at least 1 km. They were cultivated in a greenhouse with a 12/12 h day: night cycle at 27°C/22°C, located in Sichuan Normal University. After 4 months, clonal fragments of *C*. *asiatica* with first-young, second-mature, third-old, and fourth-oldest were selected. The clonal fragments were subjected to low water availability (20% volumetric soil moisture content).

### Experimental design

**Fig 1:**
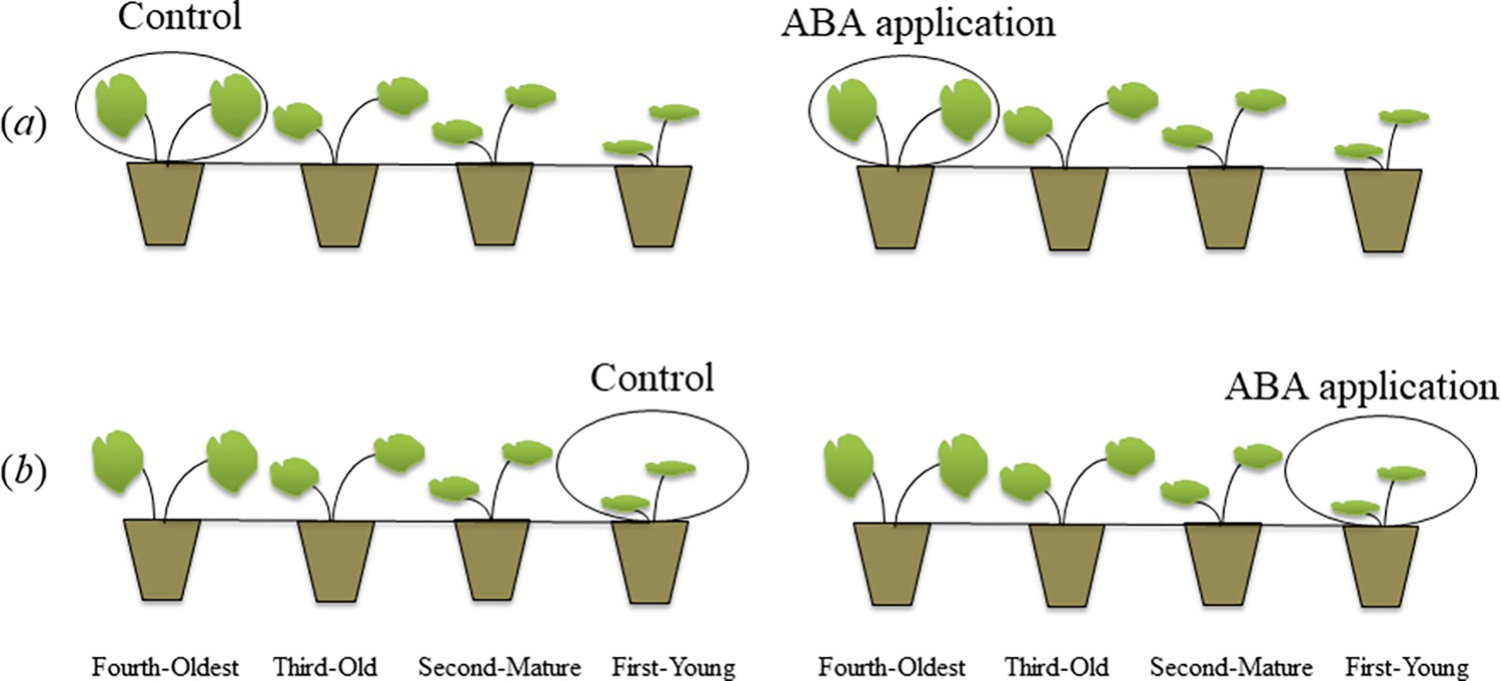
Schematic representation of the experimental design. (a): 5 mL ABA solution (0.1 mM) was applied to the fourth-oldest ramets; (b): 5 mL ABA solution (0.1 mM) was applied to first-young ramets. Same volume distilled water was employed as control.

5 mL ABA solution (0.1 mM) was applied to leaves of the first-young or fourth-oldest ramets respectively (Fig 1). As control, same volume distilled water was used [26]. Then, the first-young or fourth-oldest ramets respectively were sealed in transparent plastic bag until dry. The experiment lasted for 30 days. Each treatment was replicated 7 times.

#### Measurement of antioxidant enzyme activities, oxidative stress and osmoregulation ability

UV-spectrophotometer was used to test antioxidant enzyme (SOD, POD, CAT and APX) activities, oxidative stress (O_2_^•−^ production rate and H_2_O_2_ content) and osmoregulation ability (proline content and total soluble protein content) in root of the interconnected ramets [27–30].

#### Measurement of morphological characteristics

WinRHIZO was used to measure total length, surface area and volume in root of the interconnected ramets [31].

#### Measurement of biomass accumulation and allocation

After oven-dried at 70°C for 72 h, each clonal fragment was separated into root, leaf and stolon to weigh. Ratio of belowground to aboveground biomass was counted [32].

### Statistical analysis

Two-way analysis of variance (ANOVA) was used to investigate the effects of exogenous ABA application, ramet age and their interaction on oxidative stress (O_2_^•−^ production rate and H_2_O_2_ content), antioxidant enzyme (SOD, POD, CAT and APX) activities, osmoregulation ability (proline and soluble protein content) and morphological characteristics (total length, surface area and volume) in root of the interconnected ramets when exogenous ABA was applied to the first-young or fourth-oldest ramets respectively [33].

One-way analysis of variance (ANOVA) was employed to investigate effects of exogenous ABA application on biomass accumulation and allocation of whole clonal fragment [34].

Pearson’s correlation coefficient was employed to investigate correlation between oxidative stress (O_2_^•−^ production rate and H_2_O_2_ content) and antioxidant capacity (SOD, POD, CAT and APX activities) [35]. Significance was set at the P ≤ 0.05 level. All analyses were conducted with SPSS 21.0 software (SPSS Inc.).

## Results

### Oxidative stress in root of the interconnected ramets

**Fig 2:**
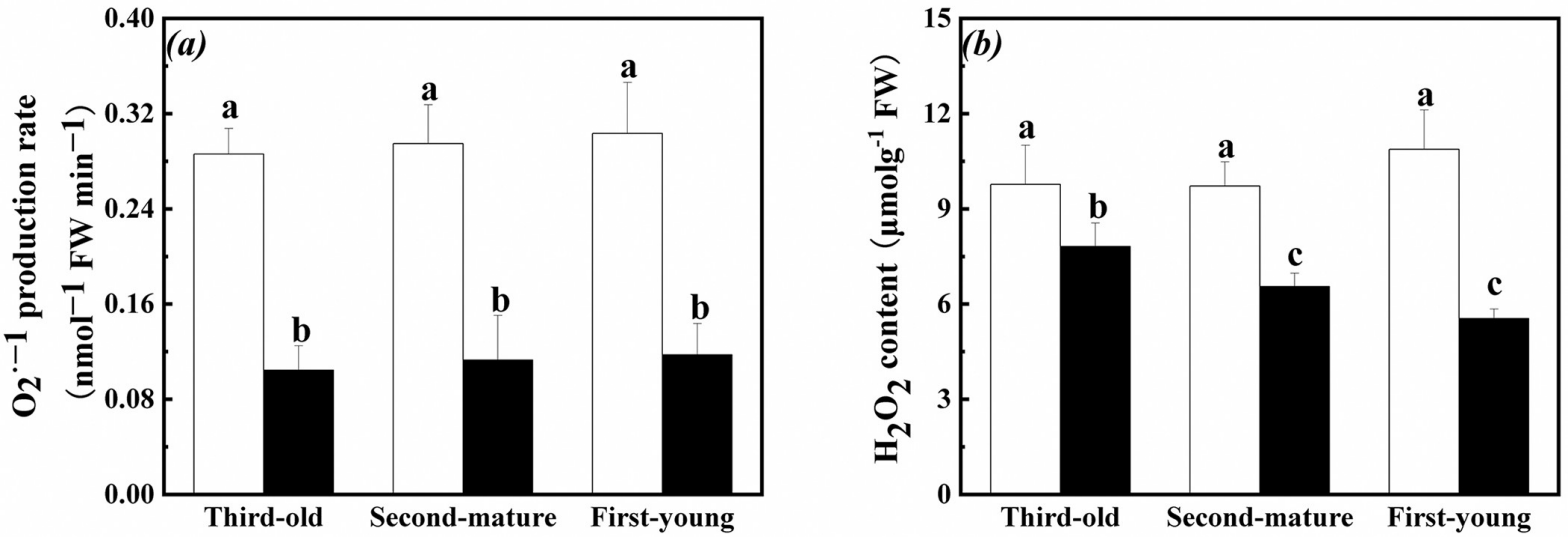
Oxidative stress in root of the first-young, second-mature and third-old ramets when exogenous ABA was applied to the fourth-oldest ramets. (a): O_2_^•−^ production rate (mean ± SE); (b): H_2_O_2_ content (mean ± SE). Open bar: control; closed bar: exogenous ABA application. Bars with the same lower case letters are not significantly different (P > 0.05).

O_2_^•−^ production rate and H_2_O_2_ content in root of the first-young, second-mature and third-old ramets significantly decreased when exogenous ABA was applied to the fourth-oldest ramets (Table 1, Fig 2A and 2B). At the same time, H_2_O_2_ content in root of the first-young and second-mature ramets significantly decreased than those in root of the third-old ramets (Table 1, Fig 2A and 2B).

**Table 1:** Effects of exogenous ABA application, ramet age and their interaction on oxidative stress (O2•− production rate and H_2_O_2_ content), antioxidant capacity (SOD, POD, CAT and APX activities), osmoregulation ability (Proline and soluble protein content), and morphological characteristics (total length, surface area and volume) in root of the first-young, second-mature and third-old ramets when exogenous ABA was applied to the fourth-oldest ramets.

Source	df	O_2_^•−^ production rate	H_2_O_2_ content	SOD	POD	CAT	APX	Proline content	Soluble protein content	Total Length	Surface area	Volume
**Exogenous ABA application**	1,36	159.693***	122.677***	208.788***	405.507***	275.093***	208.788***	151.838***	75.086***	3.378^ns^	0.773^ns^	5.611*
**Ramet age**	2.36	0.366^ns^	1.721^ns^	27.939***	29.057***	17.229***	27.939***	1.952^ns^	2.126^ns^	7.059**	3.423*	2.478^ns^
**Exogenous ABA application × ramet age**	2.36	0.01^ns^	9.802**	1.093^ns^	3.716*	21.054***	1.093^ns^	0.863^ns^	0.518^ns^	7.547**	4.848*	3.644*

***, P < 0.001

**, P < 0.01

*, P < 0.05

ns, non-significant (P > 0.05).

Abbreviations: df, degrees of freedom; SOD, superoxide dismutase; POD, peroxidase; CAT, catalase; APX, ascorbate peroxidase.

**Fig 3:**
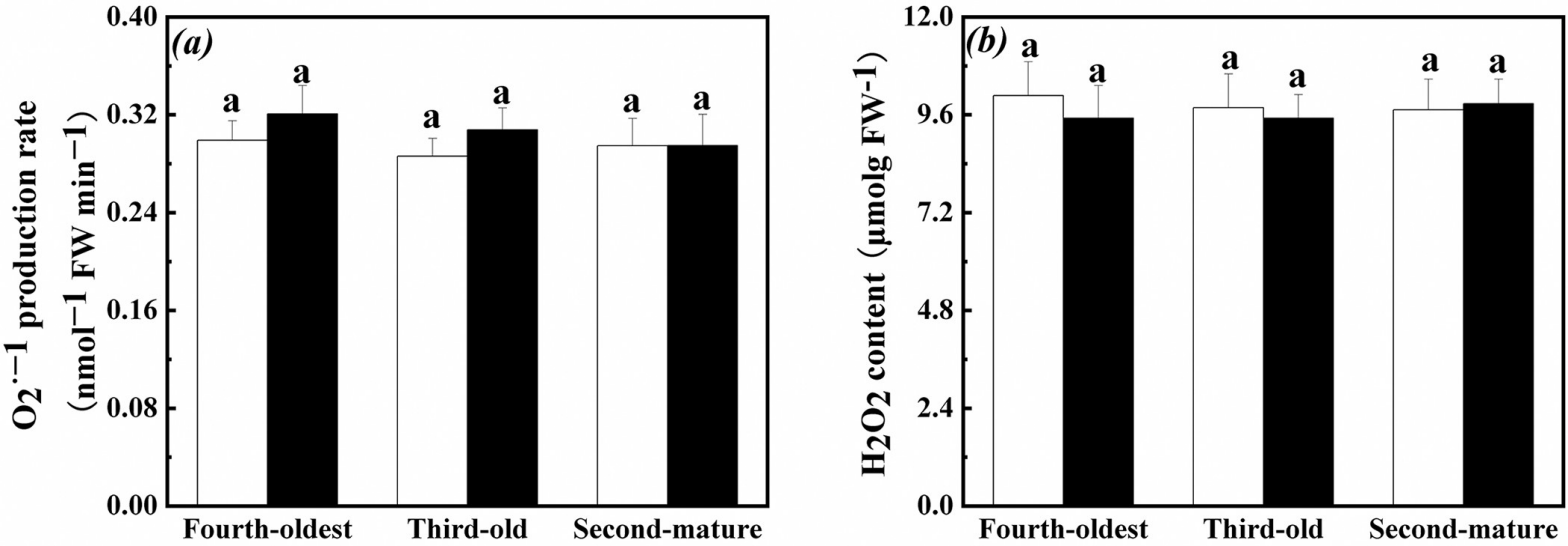
Oxidative stress in root of the second-mature, third-old and fourth-oldest ramets when exogenous ABA was applied to the first-young ramets. (a): O_2_^•−^ production rate (mean ± SE); (b): H_2_O_2_ content (mean ± SE). Open bar: control; closed bar: exogenous ABA application. Bars with the same lower case letters are not significantly different (P > 0.05).

In addition, O_2_^•−^ production rate and H_2_O_2_ content in root of the second-mature, third-old and fourth-oldest ramets were not significantly affected by exogenous ABA application, ramet age and their interaction when exogenous ABA was applied to the first-young ramets (Table 2, Fig 3A and 3B).

**Table 2:** Effects of exogenous ABA application, ramet age and their interaction on oxidative stress (O2•− production rate and H_2_O_2_ content), antioxidant capacity (SOD, POD, CAT and APX activities), osmoregulation ability (Proline and soluble protein content), and morphological characteristics (total length, surface area and volume) in root of the second-mature, third-old and fourth-oldest ramets when exogenous ABA was applied to the first-young ramets.

Source	df	O_2_^•−^ production rate	H_2_O_2_ content	SOD	POD	CAT	APX	Proline content	Soluble protein content	Total Length	Surface area	Volume
**Exogenous ABA application**	1,36	1.442^ns^	122.677***	208.788***	405.507***	275.093***	208.788***	151.838***	75.086***	3.378^ns^	2.088^ns^	1.646^ns^
**Ramet age**	2,36	0.62^ns^	0.061^ns^	2.248^ns^	2.039^ns^	0.289^ns^	6.247**	8.141**	8.832**	5.513*	5.907**	5.724**
**Exogenous ABA application × ramet age**	2,36	0.361^ns^	0.249^ns^	2.531^ns^	3.544*	0.359^ns^	1.891^ns^	0.36^ns^	8.856**	1.018^ns^	0.019^ns^	0.547^ns^

***, P < 0.001

**, P < 0.01

*, P < 0.05

ns, non-significant (P > 0.05).

Abbreviations: df, degrees of freedom; SOD, superoxide dismutase; POD, peroxidase; CAT, catalase; APX, ascorbate peroxidase.

### Antioxidant enzyme activities in root of the interconnected ramets

**Fig 4:**
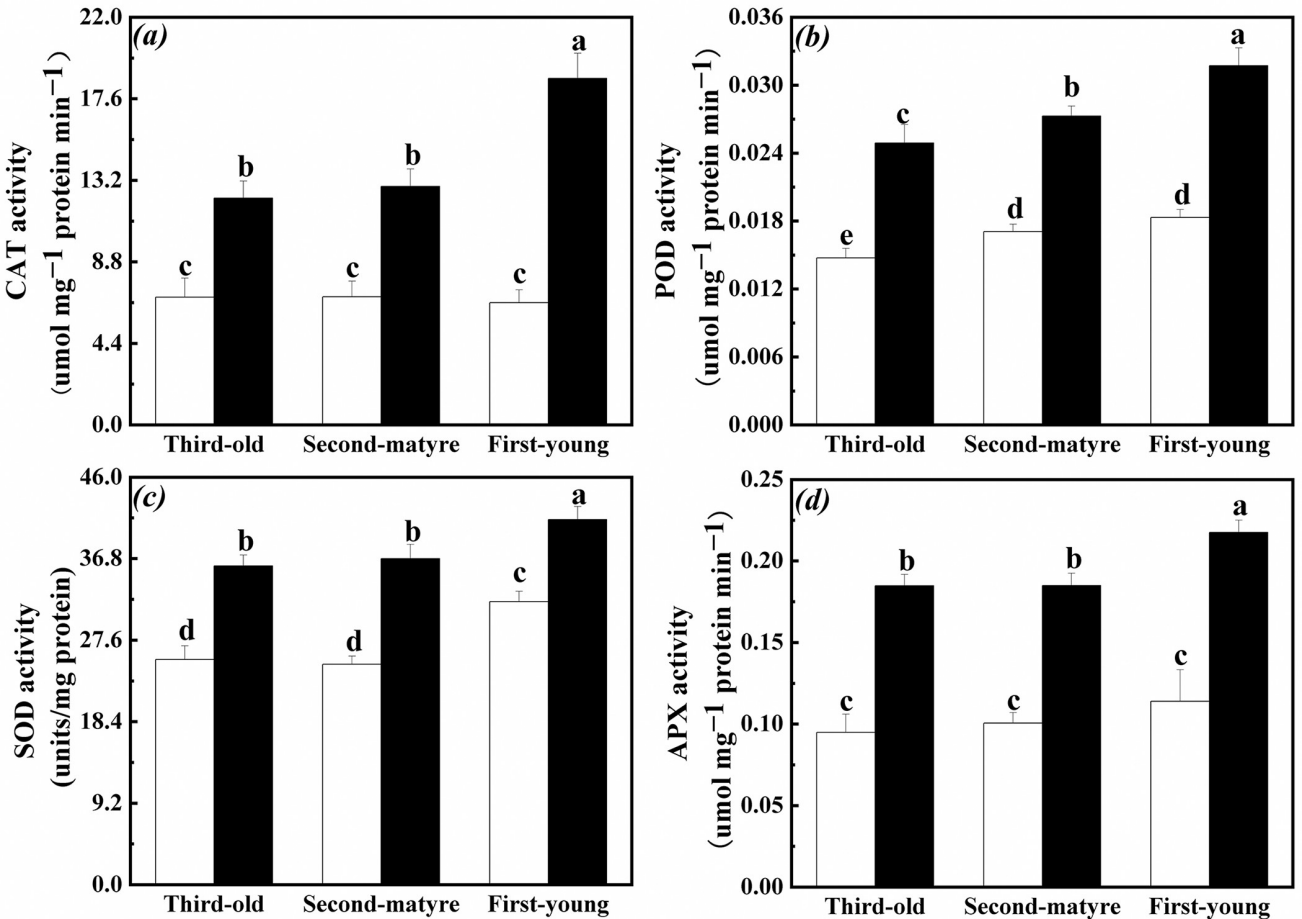
Antioxidant enzyme activities in root of the first-young, second-mature and third-old ramets when exogenous ABA was applied to the first-young ramets. (a): superoxide dismutase (SOD) (mean ± SE); (b): peroxidase (POD) (mean ± SE); (c): catalase (CAT) (mean ± SE); (d): ascorbate peroxidase (APX) (mean ± SE). Open bar: control; closed bar: exogenous ABA application. Bars with the same lower case letters are not significantly different (P > 0.05).

CAT, POD, SOD and APX activities in root of the first-young, second-mature and third-old ramets significantly increased when exogenous ABA was applied to the fourth-oldest ramets (Table 1, Fig 4A–4D). Especially, POD, CAT and APX activities in root of the first-young ramets were significantly greater than those in the root of the second-mature and third-old ramets (Table 1, Fig 4A, 4B and 4D).

Significantly positive correlation was found among the CAT, POD, SOD and APX activities in root of the interconnected ramets when exogenous ABA was applied to the fourth-oldest ramets (Table 3). Significantly negative correlation was found between H_2_O_2_ content and antioxidant enzyme (CAT, POD, SOD and APX) activities (Table 3). Similar pattern was not observed between O_2_^•−^ production rate and antioxidant enzyme (CAT, POD, SOD and APX) activities (Table 3).

**Table 3:** Correlation between oxidative stress (O2•− production rate and H_2_O_2_ content) and antioxidant capacity (SOD, POD, CAT and APX activities), in root of the first-young, second-mature and third-old ramets when exogenous ABA was applied to the fourth-oldest ramets.

Source	POD		SOD		APX		O_2_^•−^ production rate		H_2_O_2_ content	
	r	P-value	r	P-value	r	P-value	r	P-value	r	P-value
**CAT**	0.935	0.000***	0.821	0.000***	0.850	0.000***	0.156	0.500^ns^	-0.695	0.000***
**POD**			0.777	0.000***	0.724	0.000***	0.207	0.367^ns^	-0.791	0.000***
**SOD**					0.874	0.000***	0.180	0.436^ns^	-0.792	0.000***
**APX**							0.102	0.661^ns^	-0.699	0.000***
**O**_**2**_^**•−**^ **production rate**									-0.293	0.197^ns^

***, P < 0.001

**, P < 0.01

*, P < 0.05

ns, non-significant (P > 0.05).

P-value (P < 0.05) is considered to be significant; r is representing Pearson’s correlation coefficient.

**Fig 5:**
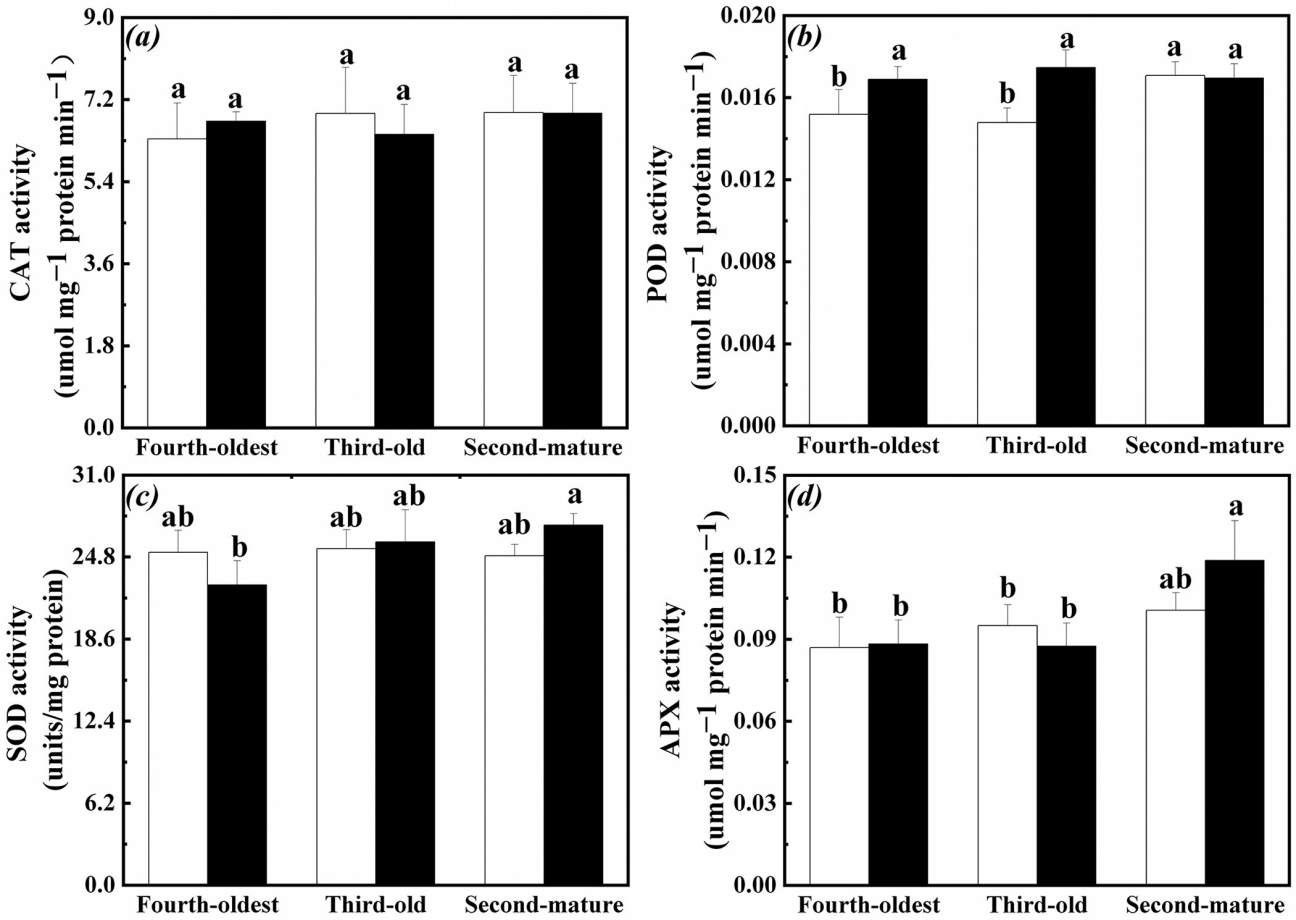
Antioxidant enzyme activities in root of the second-mature, third-old and fourth-oldest ramets when exogenous ABA was applied to the first-young ramets. (a): superoxide dismutase (SOD) (mean ± SE); (b): peroxidase (POD) (mean ± SE); (c): catalase (CAT) (mean ± SE); (d): ascorbate peroxidase (APX) (mean ± SE). Open bar: control; closed bar: exogenous ABA application. Bars with the same lower case letters are not significantly different (P > 0.05).

POD activity in root of the third-old and fourth-oldest ramets significantly increased except for that in root of the second-mature ramets when exogenous ABA was applied to the first-young ramets (Table 2, Fig 5B). At the same time, SOD activity in root of the second-mature ramets and third-old ramets significantly increased than those in root of the fourth-oldest ramets (Table 2, Fig 5C). APX activity in root of the second-mature ramets significantly increased than those in root of the third-old and fourth-oldest ramets (Table 2, Fig 5D).

CAT activity showed a positive and significant correlation with POD activity, whilst non-significant association with SOD and APX activities when exogenous ABA was applied to the first-young ramets (Table 4). At the same time, POD activity showed a positive and significant correlation with SOD activity, whilst non-significant association with APX activity (Table 4). SOD activity showed a positive but non-significant association with APX activity (Table 4). O_2_^•−^ production rate showed a negative and significant correlation with SOD activity, whilst non-significant association with CAT, POD and APX activities (Table 4). H_2_O_2_ content showed a negative and significant correlation with O_2_^•−^ production rate, whilst non-significant association with CAT, POD and APX activities (Table 4).

**Table 4:** Correlation between oxidative stress (O2•− production rate and H_2_O_2_ content) and antioxidant capacity (SOD, POD, CAT and APX activities), in root of the second-mature, third-old and fourth-oldest ramets when exogenous ABA was applied to the first-young ramets.

Source	POD		SOD		APX		O_2_^•−^ production rate		H_2_O_2_ content	
	r	P-value	r	P-value	r	P-value	r	P-value	r	P-value
**CAT**	0.634	0.002**	0.329	0.145^ns^	0.100	0.668^ns^	-0.315	0.164^ns^	0.074	0.749^ns^
**POD**			0.468	0.032*	-0.310	0.172^ns^	-0.374	0.095^ns^	0.056	0.808^ns^
**SOD**					0.373	0.096^ns^	-0.615	0.003**	0.244	0.286^ns^
**APX**							-0.121	0.600^ns^	0.098	0.672^ns^
**O**_**2**_^**•−**^ **production rate**									-0.057	0.019*

***, P < 0.001

**, P < 0.01

*, P < 0.05

ns, non-significant (P > 0.05).

P-value (P < 0.05) is considered to be significant; r is representing Pearson’s correlation coefficient.

### Osmoregulation ability in root of the interconnected ramets

**Fig 6:**
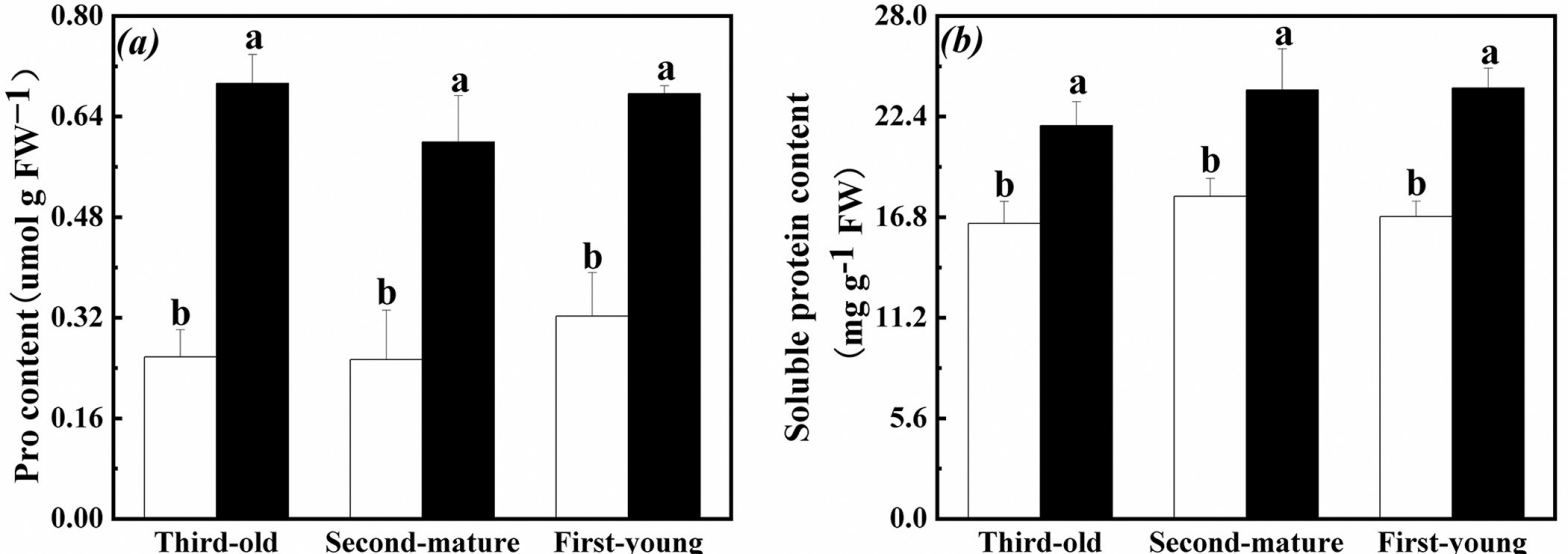
Osmoregulation ability in root of the first-young, second-mature and third-old ramets when exogenous ABA was applied to the fourth-oldest ramets. (a): proline (Pro) content (mean ± SE); (b): soluble protein content (mean ± SE). Open bar: control; closed bar: exogenous ABA application. Bars with the same lower case letters are not significantly different (P > 0.05).

Proline and soluble protein contents in root of the first-young, second-mature and third-old ramets significantly increased when exogenous ABA was applied to the fourth-oldest ramets (Table 1, Fig 6A and 6B).

**Fig 7:**
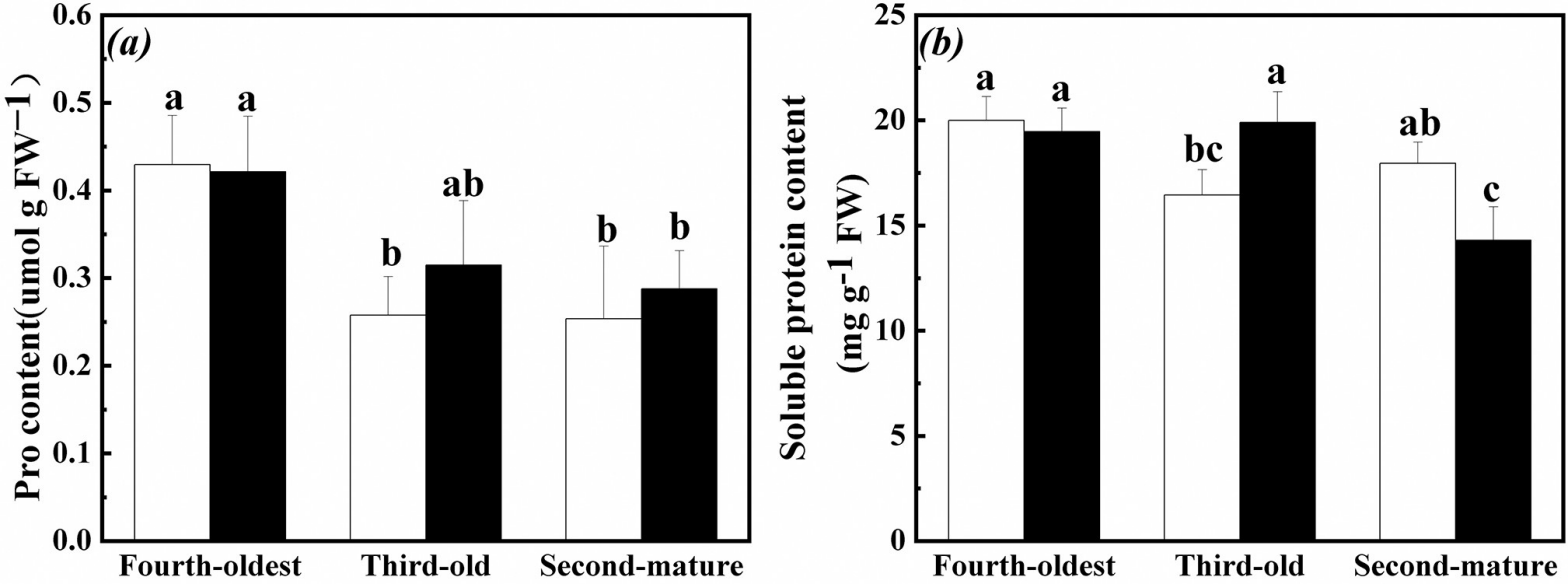
Osmoregulation ability in root of the second-mature, third-old and fourth-oldest ramets when exogenous ABA was applied to the first-young ramets. (a): proline (Pro) content (mean ± SE); (b): soluble protein content (mean ± SE). Open bar: control; closed bar: exogenous ABA application. Bars with the same lower case letters are not significantly different (P > 0.05).

Proline content in root of the second-mature, third-old and fourth-oldest ramets were not significantly effects by exogenous ABA application to the first-young ramets (Table 2, Fig 7A). In addition, soluble protein content in root of the third-old ramets significantly increased with decrease of second-mature rements when exogenous ABA was applied to the first-young ramets (Table 2, Fig 7B). Meanwhile, proline and soluble protein content in root of the second-mature ramets were lower than those of the third-old and fourth-oldest ramets (Table 2, Fig 7A and 7B).

### Morphological plasticity in root of the interconnected ramets

**Fig 8:**
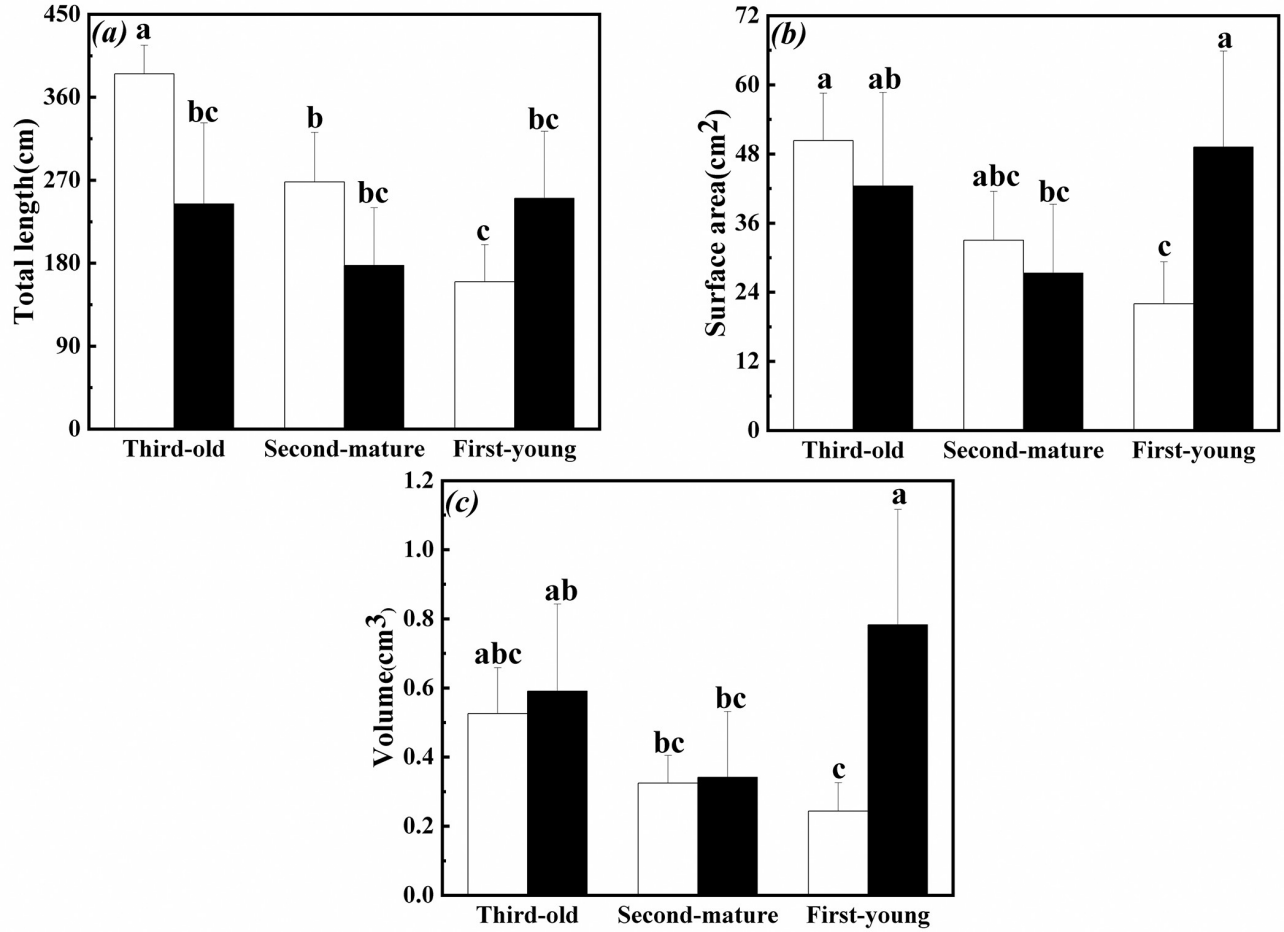
Morphological plasticity in root of the first-young, second-mature and third-old ramets when exogenous ABA was applied to the fourth-oldest ramets. (a): total length (mean ± SE); (b): surface area (mean ± SE); (c): volume (mean ± SE); Open bar: control; closed bar: exogenous ABA application. Bars with the same lower case letters are not significantly different (P > 0.05).

Total length in root of the third-old ramets significantly decreased with increase of surface area and volume in root of the first-young when exogenous ABA was applied to the fourth-oldest ramets (Table 1, Fig 8A–8C).

**Fig 9:**
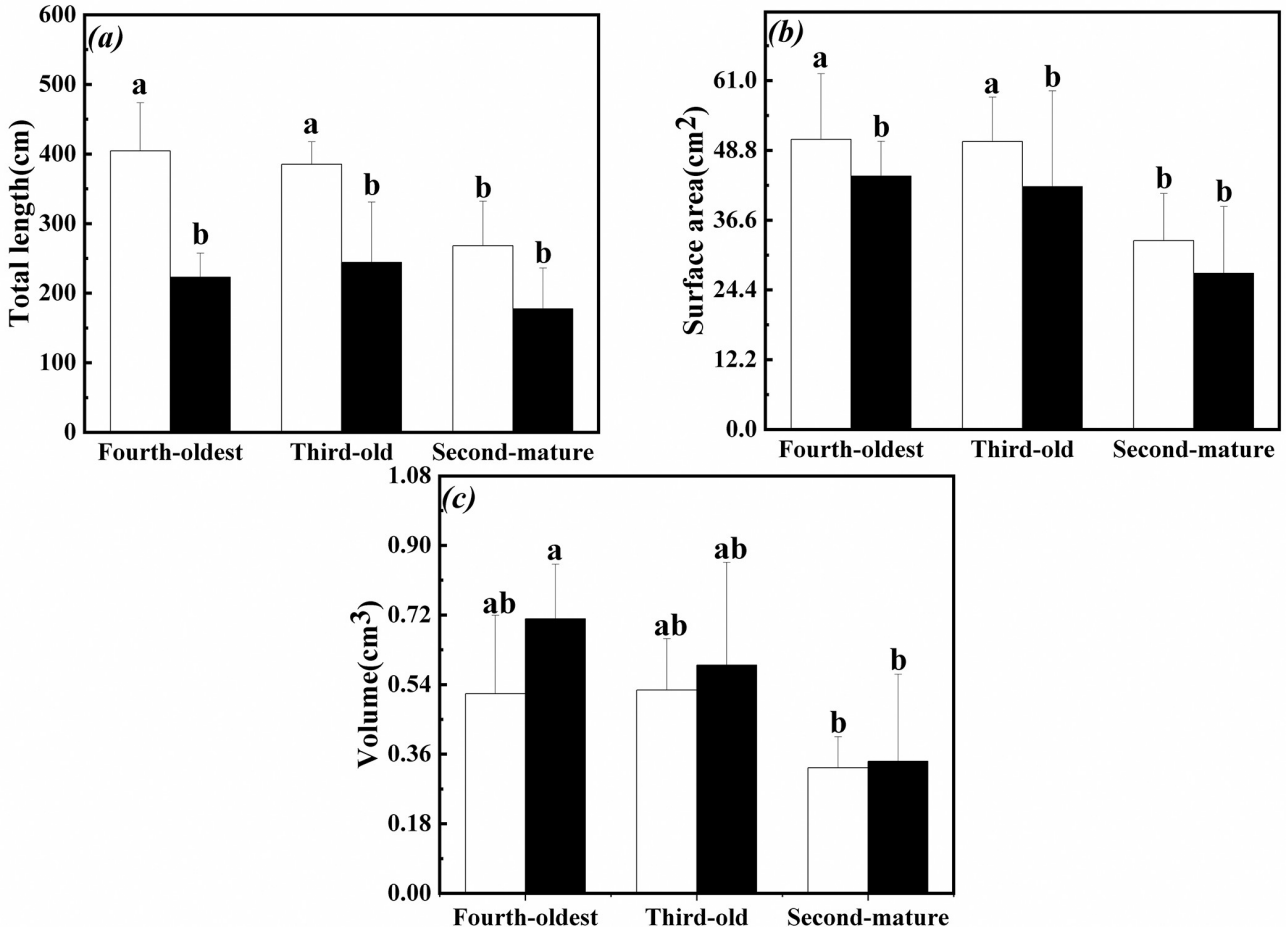
Morphological plasticity in root of the second-mature, third-old and fourth-oldest ramets when exogenous ABA was applied to the first-young ramets. (a): total length (mean ± SE); (b): surface area (mean ± SE); (c): volume (mean ± SE); Open bar: control; closed bar: exogenous ABA application. Bars with the same lower case letters are not significantly different (P > 0.05).

Total length and surface area in root of the fourth-oldest and third-old ramets significantly decreased when exogenous ABA was applied to the first-young ramets (Table 2, Fig 9A and 9B). Meanwhile, volume in root of the fourth-oldest ramets was greater than that in root of the second-mature ramets (Table 2, Fig 9C).

### Biomass accumulation and allocation in the whole clonal fragment

**Fig 10:**
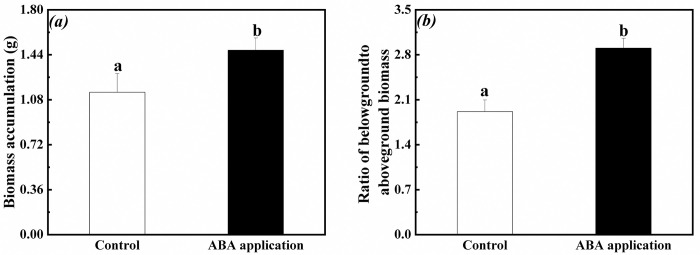
Biomass accumulation and allocation in the whole clonal fragments when exogenous ABA was applied to the fourth-oldest ramets. (a): biomass accumulation (mean ± SE), (b): ratio of belowground to aboveground biomass (mean ± SE). Open bar: control; closed bar: exogenous ABA application. Bars with the same lower case letters are not significantly different (P > 0.05).

**Fig 11:**
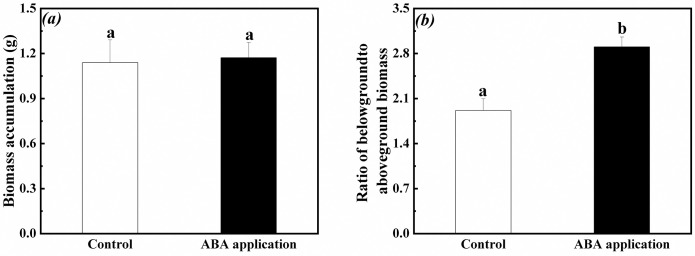
Biomass accumulation and allocation in the whole clonal fragments when exogenous ABA was applied to the first-young ramets. (a): biomass accumulation (mean ± SE), (b): ratio of belowground to aboveground biomass (mean ± SE). Open bar: control; closed bar: exogenous ABA application. Bars with the same lower case letters are not significantly different (P > 0.05).

Biomass accumulation and ratio of belowground to aboveground biomass in the whole clonal fragments significantly increased when exogenous ABA was applied to the fourth-oldest ramets (Fig 10A and 10B). Ratio of belowground to aboveground biomass in the whole clonal fragment significantly increased except for biomass accumulation when exogenous ABA was applied to the first-young ramets (Fig 11A and 11B).

In the experiment, oxidative stress in root of interconnected ramets was alleviated by increase of antioxidant enzyme activity after local exogenous ABA application (Tables 1–4, Figs 2–7). Meanwhile, change of root morphology and biomass allocation of whole clonal fragments subjected to low water availability were responsible for improving of their growth performance (Tables 1 and 2, Figs 8–11).

## Discussion

Exogenous ABA (60μM) application induced enhancement of foliar antioxidant enzyme activities (such as SOD, POD, APX, CAT) in kiwifruit subjected to drought stress, which alleviated oxidative stress (decrease of MDA and H_2_O_2_ content) in its leaf [36]. In addition, exogenous ABA application enhanced osmoregulation ability (increase of proline accumulation) in leaf of turgid barley seedlings (*Hordeum rulgare v*ar Larker) [37]. In the experiment, root systemic resistance (such as antioxidant enzyme activities and osmoregulation ability) induced by local exogenous ABA application, thereby improving growth performance of whole clonal fragments. Meanwhile, negative correlation between antioxidant enzyme (SOD, POD, CAT and APX) activities and H_2_O_2_ content in root of the first-young, second-mature and third-old ramets was observed when exogenous ABA was applied to the fourth-oldest ramets. The oxidative damage (such as H_2_O_2_) of the clonal fragments subjected to drought stress was alleviated by increase of antioxidant enzyme activity such as superoxide dismutase (SOD), catalase (CAT), peroxidases (POD), and ascorbate peroxidase (APX) [16, 38, 39].

Their root growth such as more lateral roots, greater primary root and total root length was improved when wild-type tomato (*Solanum lycopersicum* L) seedlings were suffering from severe drying [40]. Meanwhile, primary root growth in *Arachis hypogaea*, *Arabidopsis* and ‘Qingzhen 1’ apple seedlings was inhibited by exogenous ABA application [41–44]. In the experiment, local exogenous ABA application to the fourth-oldest ramets resulted in significant increase of surface area and volume in root of the first-young ramets as well as decrease of total length in root of the third-old ramets. The comprehensive effect between exogenous ABA application and drought stress on root growth may explain the above phenomena. Further, more studies are needed.

Defensive signal driven by phloem flow within clonal network mainly directed towards the acropetal direction [45, 46]. Transportation or sharing of defense signal against phloem flow was detected when the old ramets were subjected to shading within clonal network [17]. Meanwhile, transportation or sharing of stress signal against phloem flow within clonal network was observed in the experiment. Compared to PEG stress (15% polyethylene glycol), net photosynthetic rate in upland rice (resistant to drought stress) and lowland rice (susceptible to drought stress) seedlings experiencing PEG-stress was significantly increased after 48 h of exogenous ABA treatment. [47]. Thus, we tentatively concluded that rapid recovery of foliar photosynthesis could partially reverse phloem flow within clonal network when exogenous ABA was applied to the first-young ramets.

Transportation or sharing of biotic and abiotic stress signal within clonal network may be very important for survival and growth of clonal plants grown in unfavourable habitat [48, 49]. Systemic resistance and root growth stimulated by transportation or sharing of stress signal in root, thereby improving growth performance of whole clonal fragments subjected to low water availability [50]. Thus, our experiment provides insight into ecological implication on clonal integration of stress signal. Further studies are needed to understand the generality of this pattern and to assess fully the ecological advantages afforded by these features.

## Supporting information

S1 Data (XLSX)
